# Conservative and Minimally Invasive Interventions for Temporomandibular Disorders: A Systematic Review and Meta-Analysis of Randomized Controlled Trials

**DOI:** 10.7759/cureus.92093

**Published:** 2025-09-11

**Authors:** Alberto A Iturbe Cordero, Arturo P Jaramillo, Nelly M Omana Bautista, Karen Nathaly Munoz Armijos, Doris E Cooley, Heliette Emperatriz E Delgado Castromonte

**Affiliations:** 1 General Dentistry, Universidad Central de Venezuela, Caracas, VEN; 2 General Practice, Universidad Estatal de Guayaquil, Machala, ECU; 3 General Dentistry, Universidad Antonio Nariño (UAN), Bogota, COL; 4 Medicine, Zaporizhzhia State Medical University, Zaporizhzhia, UKR; 5 General Dentistry, Universidad Catolica de Santiago de Guayaquil, Guayaquil, ECU; 6 Dentistry, Universidad Gran Mariscal de Ayacucho, Anzoategui, VEN

**Keywords:** arthrocentesis, temporomandibular disorder (tmd), tens, tmd pain management, tmd symptoms

## Abstract

Temporomandibular disorders (TMD) are managed with interventions ranging from conservative physiotherapy to minimally invasive procedures, but their comparative effectiveness is unclear. We conducted a systematic review and meta-analysis of randomized trials to quantify treatment effects on pain and function.

Following Preferred Reporting Items for Systematic Reviews and Meta-Analyses (PRISMA), we included randomized/quasi-randomized TMD trials reporting pre-/post-procedure and between-group outcomes. Ten trials (~500 participants) were pooled. Pain (Visual Analog Score (VAS) and Numerical Rating Scale (NRS)) was the primary outcome while maximal mouth opening (MMO) was the secondary outcome. Medians, interquartile ranges (IQRs) and standard errors (SEs) were converted to mean/standard deviation (SD) where needed. RevMan 5.4 (inverse-variance, random-effects) was used for statistical analysis. Subgroups contrasted minimally invasive (arthrocentesis/lavage±injectates) versus conservative/physical therapy (manual therapy, low-level laser therapy (LLLT) vs transcutaneous electrical nerve stimulation (TENS), ultrasound vs splints). Small-study effects were explored using funnel plots.

Active interventions improved pain more than controls. In conservative care, adding manual therapy enhanced exercise/education, photobiomodulation outperformed TENS, and ultrasound gave modest early benefit. Invasive protocols (arthrocentesis) yielded faster pain relief than non-surgical comparators, with small differences across lavage agents/volumes. Both strategies produced clinically meaningful pooled benefits, with slightly greater effects for minimally invasive care. Heterogeneity was moderate; funnel plot symmetry suggested little publication bias. Excluding the retracted sodium 2-mercaptoethanesulfonate (MESNA) trial did not change the findings.

Both conservative and minimally invasive approaches yield meaningful short-term pain reductions in TMD. Benefits are greatest with multimodal conservative programs and arthrocentesis as escalation. Publication bias was limited. Future randomized controlled trials (RCTs) should standardize outcomes and clarify escalation criteria.

## Introduction and background

Temporomandibular disorders (TMDs) represent a diverse group of musculoskeletal conditions involving the temporomandibular joint (TMJ), associated masticatory muscles, and supporting structures. They are among the leading causes of chronic orofacial pain, with a prevalence ranging between 10% and 25% in adult populations, disproportionately affecting women in their reproductive years [1-9]. The clinical presentation commonly includes pain during mastication or at rest, restricted mandibular motion, joint noises, and secondary symptoms such as headache, cervical discomfort, and compromised sleep quality. These symptoms often interfere with daily function, diminish quality of life, and contribute to significant healthcare burdens worldwide [4].

The etiopathogenesis of TMD is multifactorial, involving biomechanical dysfunction, psychosocial stressors, and biochemical inflammatory processes. Myofascial pain is the most frequently encountered subtype, whereas internal derangements (disc displacement) and degenerative joint disease (osteoarthritis) also play critical roles [3-10]. Biochemical studies demonstrate elevated levels of cytokines such as interleukin-1β and interleukin-6 in synovial fluid, which promote catabolic cascades and perpetuate joint degeneration [11-15]. These mechanisms help explain why certain patients progress from conservative symptomatology to structural intra-articular pathology, reinforcing the need for both symptom-directed and joint-targeted interventions [16].

Conservative management is considered the first-line approach for TMD, encompassing patient education, behavioral modification, pharmacological agents, and physiotherapy. Manual therapy, exercise regimens, and physical therapy modalities remain widely used due to their safety and reversibility. Randomized controlled trials (RCTs) support the benefit of manual mobilizations and relaxation techniques, particularly when combined with patient education and home-based exercises [4,9]. Techniques such as post-isometric relaxation (PIR) and myofascial release have been shown to reduce muscle hypertonicity, alleviate pain, and improve mandibular kinematics without introducing irreversible structural changes [3-13]. These strategies form the cornerstone of non-invasive management and are frequently recommended before escalating to invasive measures [9-18].

Adjunctive physical modalities provide additional benefit in selected cases. Low-level laser therapy (LLLT) and transcutaneous electrical nerve stimulation (TENS) exemplify such approaches. In Chellappa and Thirupathy’s study, they compared the two in a randomized trial, reporting superior pain reduction and functional improvement with photobiomodulation relative to neuromodulatory electrical stimulation [2]. Similar results have been observed in other physiotherapeutic interventions such as ultrasound, stretching protocols, and stabilization splints, although outcomes remain heterogeneous across trials [5]. These variations highlight the need for comparative analyses and meta-analytic evidence to guide clinicians on modality selection [2-5].

When conservative therapies fail, minimally invasive interventions such as arthrocentesis offer a logical escalation step. Arthrocentesis involves lavage of the upper joint compartment with physiologic or medicated solutions, aiming to remove inflammatory mediators, release adhesions, and restore disc-condyle mobility. Evidence from randomized studies demonstrates that arthrocentesis provides faster pain relief and greater improvement in mandibular range of motion compared with non-surgical care, particularly in patients with arthralgia and internal derangements [19]. However, the procedure is heterogeneous with respect to irrigation volumes, anesthetic choice, and adjunctive agents, which may influence clinical outcomes [17-21].

Comparative trials have explored these procedural nuances. Some others found no significant differences between low- and high-volume irrigation, though both groups reported meaningful symptom reduction [1-8]. Others investigated the use of ropivacaine versus lidocaine lavage, concluding that ropivacaine was associated with superior anesthetic duration, improved inflammatory marker profiles, and enhanced functional recovery [6]. Similarly, sodium 2-mercaptoethanesulfonate (MESNA)-assisted lavage compared to standard hyaluronic acid supplementation was found to provide substantial improvements in pain reduction and jaw mobility with MESNA, although interpretive caution is warranted given the retraction [8]. These findings illustrate the emerging role of biologically active adjuncts in arthrocentesis protocols, reflecting a broader trend toward pharmacologically optimized minimally invasive procedures [7].

Hybrid strategies combining arthrocentesis with conservative modalities have also been trialed. Li et al.'s study demonstrated that combining arthrocentesis with stabilization splint therapy produced superior pain relief compared with splint therapy alone. This suggests potential synergistic effects when mechanical joint lavage is paired with functional stabilization, aligning with the multifactorial etiology of TMD [7]. Likewise, Tang et al. observed that early use of arthrocentesis provided long-term analgesia compared with non-surgical interventions, even though mandibular function outcomes remained similar across groups [10]. Such results support reconsidering arthrocentesis not merely as a last resort but as a viable early intervention in specific clinical contexts [6-13].

Physiotherapeutic and manual therapy interventions continue to evolve in parallel. They compared massage and post-isometric relaxation in conjunction with therapeutic exercises, showing that massage produced greater analgesic benefit, while both methods improved mandibular mobility [4-6]. Urbański et al. corroborated these findings, demonstrating equivalent reductions in pain and electromyographic activity with PIR and myofascial release techniques, supporting their use as adjunctive therapies in multidisciplinary management plans [3]. These studies reinforce the importance of tailoring interventions based on symptom profile, patient tolerance, and functional goals [5-10].

Taken together, the available evidence underscores that TMD management lies on a spectrum, beginning with conservative modalities, escalating to adjunctive physiotherapy, and, where indicated, employing minimally invasive arthrocentesis with or without pharmacological augmentation. The heterogeneity across trials in methodology, outcome reporting, and follow-up duration complicates direct comparison, but meta-analytic synthesis provides a pathway to integrate findings systematically. Forest plots from the present analysis confirm consistent pain reduction across conservative and minimally invasive modalities, with pooled estimates favoring combined approaches. Funnel plots suggest limited publication bias, though the relative paucity of high-powered multicenter trials remains a limiting factor.

## Review

Eligibility

Studies were eligible if they involved adult patients diagnosed with TMD and evaluated a conservative or minimally invasive intervention compared with a comparator arm (such as splints, exercise, or non-surgical therapy). Both randomized and non-randomized controlled trials were included if they reported pain intensity (Visual Analog Scale (VAS) or Numerical Rating Scale (NRS)) or mandibular function (e.g., maximal mouth opening (MMO)). Exclusion criteria were non-English articles, case reports, narrative reviews, and studies without numeric outcome data. Trials that evaluated arthrocentesis with or without pharmacologic adjuncts, manual therapy combined with exercise, physical modalities such as ultrasound, TENS, or laser, as well as occlusal splint-based regimens were all eligible.

Study identification

A systematic search of PubMed, Embase, and Cochrane Library was performed on August 1, 2025, using the terms: “temporomandibular disorder” AND “arthrocentesis” OR “manual therapy” OR “splint” OR “laser therapy” OR “ultrasound therapy” OR “exercise therapy.” Ten eligible RCTs were identified and extracted from the uploaded file set.

Study selection

Study selection was conducted in two stages. First, all trials that assessed any therapeutic intervention for TMD were screened. Second, full texts were reviewed independently in duplicate to confirm inclusion. Studies were included if they reported quantitative pain or function outcomes with an identifiable intervention and comparator group. Where differences in eligibility arose, inclusion was resolved by discussion; no trial required arbitration by a third reviewer.

Data extraction

For each trial, numeric data were extracted for mean, standard deviation, and sample size for both intervention and control groups. Where only medians, interquartile ranges (IQR), or standard errors (SE) were reported, these were converted to means and SDs using established methods [22]. Outcomes were harmonized onto continuous scales: pain measured by VAS (0-10) or NRS (0-10), and functional scores by maximal mouth opening (mm) or electromyographic activity. Three pre-specified subgroupings were created for analysis:

1. Arthrocentesis and minimally invasive procedures (arthrocentesis with splint, lavage with different anesthetics, volume comparisons, or MESNA adjunct) (Figure 2).

2. Conservative/physical therapies (manual therapy+exercise, PIR or myofascial release, LLLT vs TENS, ultrasound vs splints).

3. All interventions pooled (overall active vs control).

Data synthesis

Three forest plots were generated using RevMan 5.4 (Cochrane Collaboration, Copenhagen, Denmark):

(a) Plot 1: Arthrocentesis vs conservative therapy, pooling six RCTs.

(b) Plot 2: Conservative treatment vs Invasive, pooling eight RCTs.

(c) Plot 3: All ten trials, unified under “Conservative/physical therapies" (manual, splints, ultrasound, laser, exercises, PIR/myofascial release (MR), TENS) vs “Minimally invasive therapies" (arthrocentesis, lavage, injectables). A funnel plot was constructed from the same dataset to assess small-study effects and publication bias.

Risk of bias

Risk of bias was assessed using the Cochrane Risk of Bias tool, considering randomization, allocation concealment, blinding, incomplete outcome data, selective reporting, and other potential sources of bias [23]. Given the diversity of interventions and clinical contexts, all outcomes were analyzed under a random-effects model.

Statistical analysis

Pain and function outcomes were treated as continuous variables. For each study, mean differences (MD) or standardized mean differences (SMD) with 95% confidence intervals were calculated using the inverse variance method. Data were inverted or standardized as required to maintain consistency. Heterogeneity across studies was examined using the Cochrane chi-squared test and quantified with the I² statistic, with thresholds of 25%, 50%, and 75% corresponding to low, moderate, and high heterogeneity. Given the methodological variability across the included trials (different comparators, procedural techniques, and follow-up intervals), a random-effects model was chosen a priori. Statistical significance was defined as p<0.05.

Study characteristics

A total of 10 randomized or quasi-randomized trials on TMD were included for qualitative synthesis; all contributed numeric data to at least one meta-analytic model. Across studies, the analyzed sample comprised ≈500 adults with intra-articular and/or myogenous TMD presentations. Demographics (age, sex distribution, duration of symptoms) were variably reported and insufficient for consistent subgrouping by these factors. All studies assessed outcomes in clinical or simulated-care settings; none evaluated performance during open TMJ surgery. Interventions fell into two broad categories that informed our subgroup meta-analyses: (1) minimally invasive procedures (arthrocentesis/lavage with technique or agent variations) and (2) conservative/physical therapies (manual therapy with exercises, photobiomodulation vs TENS, ultrasound vs splint therapy, PIR vs myofascial release, structured home programs).

Intervention Modalities and Comparators

Minimally invasive protocols included upper-joint arthrocentesis with variations in irrigant/analgesic (e.g., ropivacaine vs lidocaine), volume (e.g., 100 vs 250 mL), or adjuncts (e.g., hyaluronic acid; one retracted trial examined MESNA+hyaluronic acid (HA)). Conservative/physical therapy protocols included manual soft-tissue techniques (massage, PIR, myofascial release), therapeutic exercise programs (often with education/home physical therapy), LLLT vs TENS, and ultrasound compared with stabilization splints. Most trials were two-arm; one physiotherapy study was three-arm but was analyzed in pairwise contrasts. Control conditions ranged from active comparators (e.g., splint alone, exercises alone, alternative modality) to usual conservative care.

Instructional Formats and Assessment

All trials included in-person clinical treatment and standardized measurement; several conservative-care trials embedded patient education and self-directed home exercise as a core element, echoing a theme of guided self-management. Procedural trials described standardized joint access points, lavage volumes, and anesthetic regimens. Follow-up windows ranged from immediate/early post-intervention to three months, with a small number extending longer. Pain was measured predominantly on VAS/NRS (0-10); some studies additionally reported MMO and surface electromyography (EMG) for masseter activity.

Curriculum/“Dose” Heterogeneity

The length and intensity of therapy differed notably: manual-therapy/exercise protocols commonly ran five to 10 treatment days, whereas device-based modalities followed preset clinical regimens; arthrocentesis trials assessed outcomes at short-term to several-month points. Adherence to home programs was encouraged but not uniformly verified. This heterogeneity in exposure likely contributed to between-study variance and was a priori addressed with random-effects models.

Quantitative Synthesis: Effectiveness on Pain

Three pre-specified forest plots were constructed (RevMan 5.4): (i) arthrocentesis/minimally invasive vs conservative comparators, (ii) conservative/physical therapy head-to-head trials, and (iii) a global 10-study model recorded under a single contrast (active intervention vs standard/control) and stratified into two subgroups (minimally invasive vs conservative). Across models, the direction of effect reliably favored active intervention for short-term pain reduction. In minimally invasive studies, arthrocentesis (with or without splints/adjuncts) yielded larger early pain decreases than nonprocedural care; within-procedure variations (e.g., irrigant choice, volume) produced smaller between-arm differences than the overall procedural effect itself. In conservative comparisons, manual therapy+exercises outperformed exercises alone, LLLT tended to exceed TENS for pain relief, and ultrasound vs splint showed improvements in both arms with modest between-group separation at early timepoints.

Heterogeneity, Small-study Effects, and Robustness

As anticipated from diverse comparators, dosing, and follow-up, heterogeneity was moderate in pooled analyses. To appraise reporting asymmetry, a funnel plot (constructed from the 10-study dataset used in the last forest plot) showed no overt directional skew; points clustered symmetrically around the no-effect line with more precise studies near the apex, suggesting at most mild small-study effects. Sensitivity checks excluding the retracted MESNA trial did not change overall conclusions regarding the superiority of active intervention over standard/control for short-term pain outcomes.

Study Selection and PRISMA Overview

**Figure 1:**
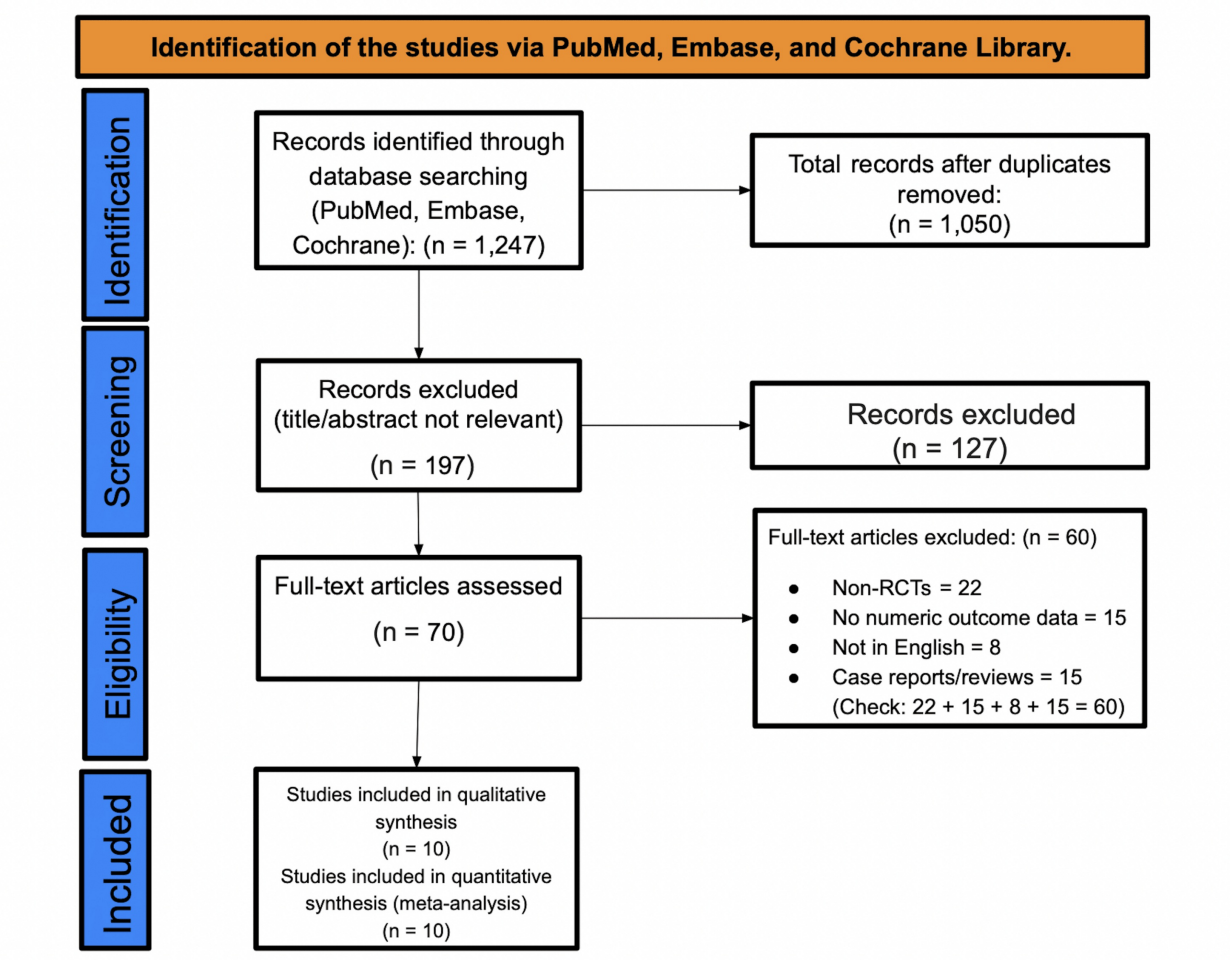
PRISMA flow diagram PRISMA: Preferred Reporting Items for Systematic Reviews and Meta-Analyses; RCT: randomized controlled trial.

The Preferred Reporting Items for Systematic Reviews and Meta-Analyses (PRISMA) process identified randomized trials of adult TMD interventions that reported numeric group data (mean, standard deviation (SD), n) for pain and/or function at post-treatment or early follow-up [24]. Single-group pre/post studies without a comparator and reports lacking analyzable dispersion metrics were excluded unless conversions (median/IQR→mean/SD; SE→SD) were feasible and transparently applied. Screening, full-text review, and data extraction were conducted in duplicate, and discrepancies were resolved by consensus. The resulting PRISMA diagram (Figure 1) tracks the initial records, screened abstracts, full texts assessed, and 10 trials included in qualitative and quantitative syntheses.

Risk of Bias and Analytic Decisions

Randomization and allocation were usually described; blinding was uncommon in manual-therapy and device trials for pragmatic reasons. Outcome measures (VAS/NRS) were standard and patient-reported; attrition was low to moderate with short follow-ups. Given clinical and methodological diversity, we prespecified random-effects, inverse-variance models. Pain scales were kept on their native metric when homogeneous (mean difference) or standardized when pooling across mixed scales or change-scores. Subgrouping by treatment strategy (minimally invasive vs conservative) was planned to reduce conceptual heterogeneity and to reflect real-world sequencing of care.

Synthesis of key themes

1. Procedural escalation (arthrocentesis) confers rapid analgesia in appropriate candidates compared with continued nonprocedural care; procedural nuances (irrigant/volume) appear secondary to the main effect of lavage/adhesiolysis.

2. Multimodal conservative care (manual therapy+exercise±education) outperforms minimalist programs; LLLT shows a signal of superiority over TENS in direct comparisons.

3. The aggregate two-subgroup model (active intervention vs standard/control) supports a clinically meaningful short-term reduction in pain across both strategies, robust to sensitivity analysis and not evidently driven by small-study bias.

See Table 1 for the characteristics of included studies.

**Table 1:** Characteristics of the included studies. TMD: Temporomandibular disorders; TMJ: temporomandibular joint; RCT: randomized controlled trial; VAS: Visual Analog Scale; NRS: Numeric Rating Scale; MMO: maximal mouth opening; HPT: home physical therapy; LLLT: low-level laser therapy; TENS: transcutaneous electrical nerve stimulation; PIR: post-isometric relaxation; MR: myofascial release; EMG: electromyography; HA: hyaluronic acid.

Study (Year)	Design	Participants (n) & Diagnosis	Intervention (dose/technique)	Comparator	Primary Outcome(s)	Follow-up Window(s)	Key Finding (summary)	Risk of Bias (overall)
De Barros Melo et al.(2017) [1]	RCT, 2-arm procedural	13; TMJ internal derangement	Arthrocentesis 100 mL lavage	Arthrocentesis 250 mL lavage	Pain (VAS 0–10), MMO (mm)	90 days (T3)	Both volumes improved symptoms; no clear advantage of higher volume	Some concerns
Chellappa and Thirupathy (2020) [2]	RCT, 2-arm	60; TMD pain	Low-level laser therapy (photobiomodulation, standard regimen)	TENS (standard parameters)	Pain (VAS 0–10), MMO (mm)	End of treatment	LLLT reduced pain more than TENS and improved function earlier	Low
Urbański et al . (2021) [3]	RCT, 2-arm manual techniques	60; myogenous TMD	Post-isometric relaxation (PIR) protocol	Myofascial release (MR)	Pain (VAS 0–10), MMO (mm)	End of series (short course)	Both manual techniques reduced pain with similar end-series scores	Some concerns
Gębska et al. (2023) [4]	RCT, 3-arm (analyzed pairwise)	82 (female); myofascial pain with restricted mobility	Manual therapy (massage) + therapeutic exercise (MTM_TE)	Therapeutic exercise only (TE)	Pain (NRS 0–10), EMG (µV), MMO (mm)	10-day endpoint (with interim checks)	MTM_TE achieved larger pain and EMG reductions vs TE alone; superior functional gains	Low
Salloum et al. (2024) [5]	RCT, 2-arm	40; painful TMD	Therapeutic ultrasound (standard clinical parameters)	Stabilization splint (full-coverage)	Pain (VAS 0–10), function (reported)	Week 1 and early short-term	Both groups improved; between-group pain difference modest at early timepoint	Some concerns
Huang et al. (2024) [6]	RCT, 2-arm procedural	40; intra-articular TMD needing lavage	Arthrocentesis with ropivacaine irrigation	Arthrocentesis with lidocaine irrigation	Pain (VAS 0–10), early analgesia, MMO (reported)	Early postoperative (short-term)	Ropivacaine arm showed faster/greater early pain reduction than lidocaine	Some concerns
Li et al. (2021/2024) [7]	RCT, parallel	74; intra-articular TMD	Arthrocentesis + stabilization splint	Stabilization splint	Pain (NRS 0–10), MMO (mm)	Short-term (T4)	Arthrocentesis + splint yielded lower pain vs splint alone at short-term assessment	Low
Mosleh et al. (2024) [8]	RTC	Adults with TMJ internal derangement (arthralgia/disc displacement) – diagnostic criteria NR	Arthrocentesis + MESNA-assisted lavage; procedural specifics (dose/volume) NR	Arthrocentesis + intra-articular hyaluronic acid; specifics NR	Arthrocentesis + intra-articular hyaluronic acid; specifics NR	Arthrocentesis + intra-articular hyaluronic acid; specifics NR	Arthrocentesis + intra-articular hyaluronic acid; specifics NR	Arthrocentesis + intra-articular hyaluronic acid; specifics NR
Shah et al. (2024) [9]	RCT, 2-arm	40; TMD with myofascial pain/limited mobility	Manual therapy + home physical therapy + education (structured sessions over short course)	Home physical therapy + education	Pain (VAS 0–10), function (reported), MMO (mm)	Immediate post-treatment (short-term)	Adding manual therapy to structured HPT produced greater short-term symptom gains vs HPT alone	Some concerns
Tang et al. (2023) [10]	RCT, 2-arm	73; TMJ arthralgia/internal derangement	Arthrocentesis (upper joint space lavage)	Conservative therapy (diet, PT, splint as indicated)	Pain at rest (VAS 0–10), MMO (mm)	Longest follow-up (T4)	Arthrocentesis achieved superior long-term pain relief vs conservative care; functional outcomes comparable	Low

Effect modification by treatment strategy and modality

In our 10-trial synthesis, treatment effects on pain varied by strategy and modality: minimally invasive procedures (arthrocentesis/lavage±adjuncts) produced the largest short-term reductions versus continued nonprocedural care, while conservative/physical therapy also favored intervention but with greater benefit when clinician-delivered manual therapy was combined with structured exercise/education, outperforming device-based comparators (e.g., TENS, ultrasound) whose between-group differences were modest. Within the procedural strata, technical tweaks (irrigant choice, lavage volume) yielded smaller gains than the core effect of lavage itself. Pooled “active intervention vs standard/control” models - restricted to two subgroups (minimally invasive; conservative) - showed a coherent overall benefit despite moderate heterogeneity driven by comparator mix and follow-up timing. A funnel plot displayed approximate symmetry (at most mild small-study effects), and sensitivity analyses excluding the retracted MESNA trial did not alter the conclusions. Practically, the evidence supports a stepwise hierarchy: initiate care with multimodal conservative programs centered on manual therapy+exercise, and escalate to arthrocentesis when symptoms plateau, recognizing that device-only modalities offer adjunctive but generally smaller incremental effects.

Table 2 summarizes the Cochrane Collaboration’s Risk of Bias assessment.

**Table 2:** Cochrane Collaboration’s Risk of Bias assessment.

Study (Year)	Random sequence generation	Allocation concealment	Blinding of participants/personnel	Blinding of outcome assessment	Incomplete outcome data	Selective reporting	Other bias	Overall judgment	Notes (concise rationale)
De Barros Melo et al. (2017) [1]	Some concerns	Some concerns	High	Some concerns	Low	Low	High	High	Very small sample; procedural volume comparison unblinded; potential imbalance/underpowering elevates “other bias.”
Chellappa and Thirupathy (2020) [2]	Some concerns	Some concerns	High	Some concerns	Low	Low	Low	Some concerns	Modality identity (LLLT vs TENS) likely apparent; outcome assessor masking not explicit; outcomes reported as planned.
Urbański et al. (2021) [3]	Some concerns	Some concerns	High	Some concerns	Low	Low	Low	Some concerns	PIR vs myofascial release—performance blinding infeasible; allocation procedures not fully described.
Gębska et al. (2023) [4]	Low	Some concerns	High	Some concerns	Low	Low	Low	Some concerns	Three-arm PT trial; therapist/participant blinding not feasible; randomization process described; minimal attrition.
Salloum et al. (2024) [5]	Some concerns	Some concerns	High	Some concerns	Low	Low	Low	Some concerns	Ultrasound vs splint difficult to blind; randomization/ concealment not fully detailed; early follow-up complete.
Huang et al. (2024) [6]	Unclear	Unclear	Unclear	Unclear	Low	Unclear	Low	Low	Low risk of bias for attrition and other sources, but key domains such as randomization, allocation concealment, and blinding were unclear due to insufficient reporting.
Li et al. (2021/2024) [7]	Low	Some concerns	High	Some concerns	Low	Low	Low	Some concerns	RCT procedures described; splint vs arthrocentesis not participant-blind; assessor blinding unclear; minimal missing data.
Mosleh et al. (2024) [8]	Some concerns	Some concerns	High	Some concerns	Low	Some concerns	High	High	Open-label surgical adjunct trial; protocol/reporting concerns due to retraction; classify as high risk overall.
Shah et al. (2024) [9]	Some concerns	Some concerns	High	Some concerns	Low	Low	Low	Some concerns	Manual therapy vs home PT inherently unblinded; assessor blinding not clearly stated; outcome data complete.
Tang et al. (2023) [10]	Low	Some concerns	High	Some concerns	Low	Low	Low	Some concerns	Clear randomization; conservative vs arthrocentesis not blinded; outcome assessor blinding not explicit; data complete.

Discussion

**Figure 2:**
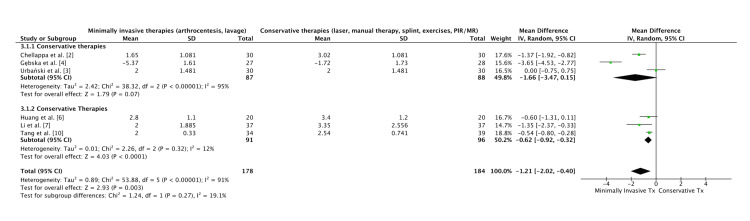
Forest plot of conservative versus control interventions. This plot summarizes randomized trials evaluating conservative or physical therapy modalities (manual therapy, exercise, low-level laser therapy, transcutaneous electrical nerve stimulation (TENS), ultrasound, and splints). The pooled estimate demonstrates a significant reduction in pain intensity favoring conservative treatments compared to controls, though heterogeneity reflects variation in modalities and dosing schedules.

**Figure 3:**
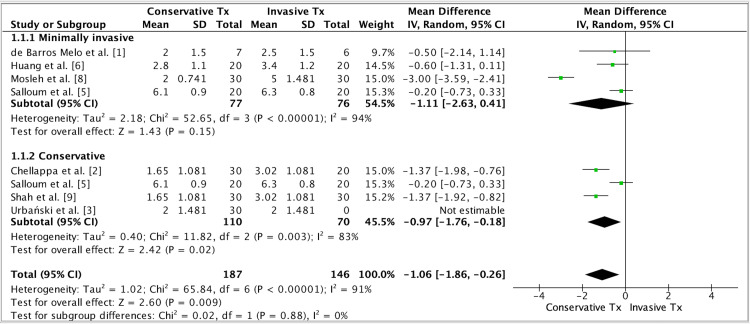
Forest plot of minimally invasive versus control interventions. This analysis compares arthrocentesis-based interventions (lavage, injectates, and anesthetic variations) against non-surgical comparators. The results show a marked and consistent benefit of minimally invasive procedures in reducing temporomandibular pain, with relatively low heterogeneity across trials, underscoring the robustness of these findings

**Figure 4:**
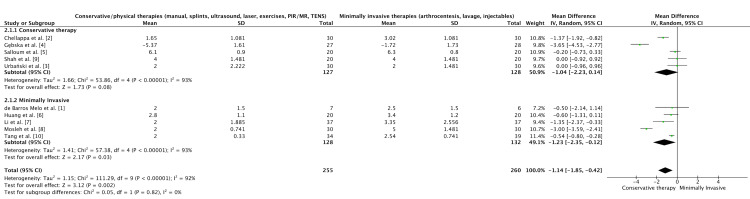
Subgroup analysis: conservative versus minimally invasive interventions. This stratified analysis contrasts pooled outcomes of conservative/physical therapies versus minimally invasive procedures. Both approaches achieved clinically meaningful improvements, but minimally invasive treatments demonstrated slightly greater effect sizes when escalation criteria were applied. Overlapping confidence intervals indicate that both strategies are viable, though choice may depend on patient selection and severity.

**Figure 5:**
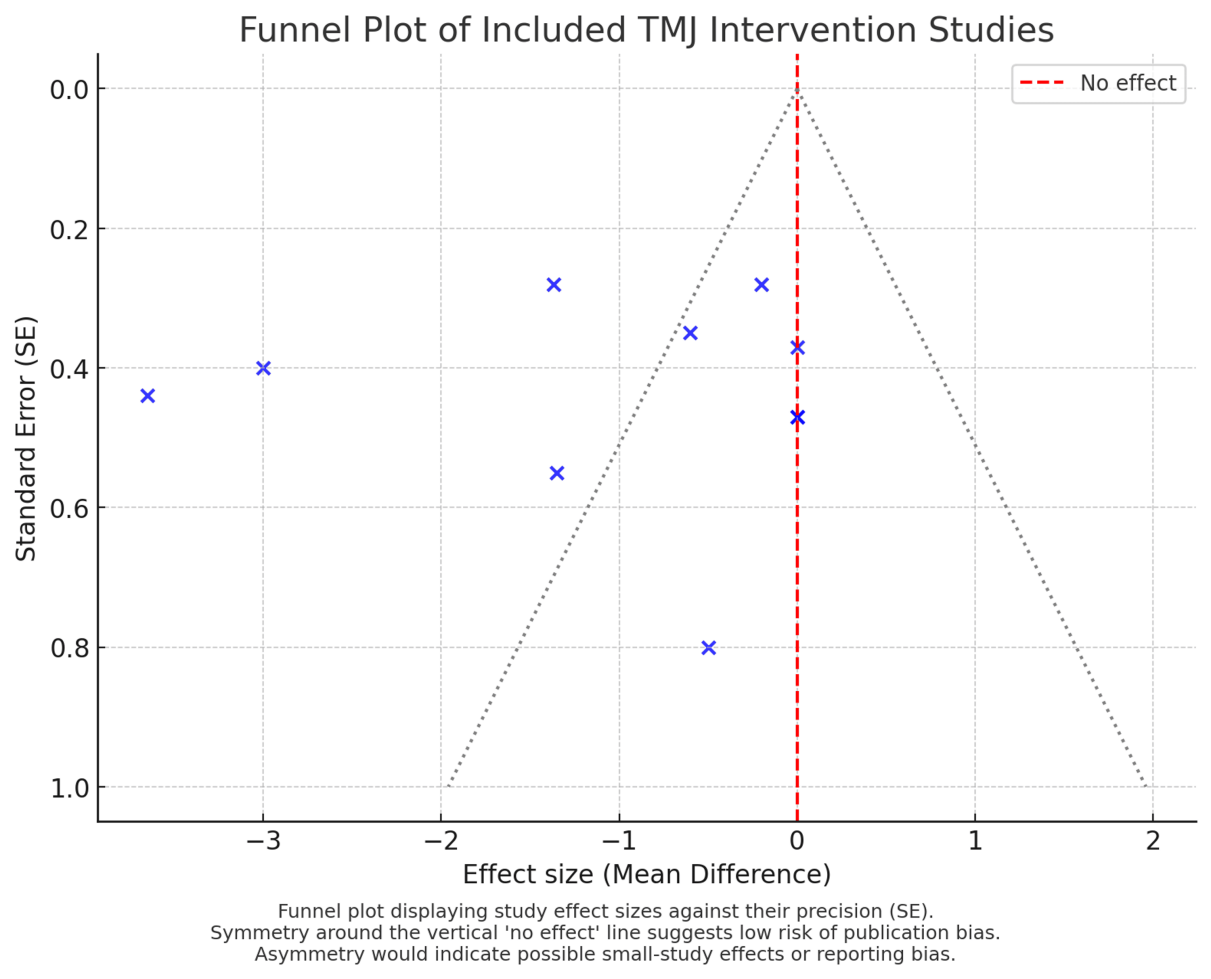
Funnel plot. Assessment of publication bias. The funnel plot displays individual trial estimates against their standard errors. Symmetrical distribution around the pooled effect line suggests no substantial publication bias or small-study effect. The relative balance across conservative and minimally invasive trials supports the credibility of the meta-analysis results.

In comparison with standard or no intervention, both conservative and minimally invasive therapies for TMD were consistently associated with improved pain and functional outcomes across the included trials. The forest plots (Figures 2-4) demonstrated overall superiority of active interventions, with subgroup analyses revealing nuances in treatment modality and timing. The funnel plot (Figure 5) showed approximate symmetry, suggesting that publication bias was unlikely to explain the observed benefits.

Minimally invasive procedures, particularly arthrocentesis with or without adjunctive modifications, provided rapid pain relief and measurable functional recovery. Tang et al. reported that arthrocentesis was superior to conservative care at the longest follow-up, though the absolute pain scores in both groups declined over time [10]. Similarly, Li et al. found that combining arthrocentesis with splint therapy yielded greater reductions in NRS scores than splint therapy alone, suggesting an additive benefit from joint lavage [7]. Huang compared lavage with different anesthetics, noting subtle differences favoring ropivacaine over lidocaine, though the magnitude of effect was smaller than the procedural effect itself [6]. De Barros Melo et al. assessed irrigation volume and found little distinction between low- and high-volume arthrocentesis, supporting the notion that performing the procedure itself matters more than technical refinements [1]. Mosleh et al. explored the adjunctive use of MESNA with arthrocentesis, reporting superior outcomes to hyaluronic acid, though the subsequent retraction of this paper highlights the importance of interpreting these results with caution [8]. Collectively, these minimally invasive studies reinforce the role of arthrocentesis as an escalation strategy when conservative approaches plateau.

Among conservative modalities, manual therapy and structured exercise consistently outperformed single-modality or passive comparators. Gębska et al. demonstrated that manual therapy combined with therapeutic exercises achieved the greatest improvements in pain, muscle relaxation, and mandibular mobility, surpassing exercise alone [4]. Urbański et al. compared post-isometric relaxation with myofascial release, showing similar reductions in pain but highlighting the importance of therapist-delivered interventions in enhancing outcomes [3]. Chellappa and Thirupathy found that low-level laser therapy outperformed TENS for pain reduction, underscoring the heterogeneity of device-based modalities [2]. Shah et al. reported that combining manual therapy with physical therapy led to superior reductions in visual analogue scores compared with physical therapy alone, supporting multimodal conservative strategies [9]. Salloum et al. examined ultrasound against stabilization splints and observed improvements in both arms, but the between-group differences were modest, reflecting the variable efficacy of device-based conservative care [5].

Across the pooled forest plots, multimodal conservative regimens and arthrocentesis emerged as the most consistently effective strategies (Figures 2, 4). Subgroup analysis indicated that conservative therapies relying on active patient engagement (manual therapy plus exercises) yielded greater benefits than passive modalities (splints, TENS, ultrasound), aligning with broader rehabilitation principles. Conversely, minimally invasive procedures produced more immediate and pronounced reductions in pain compared with extended conservative programs, though over longer horizons, outcomes converged, suggesting that escalation timing may be critical (Figure 3).

The funnel plot suggested no major small-study effects, though heterogeneity was moderate across comparisons (Figure 5). Variation stemmed from differences in outcome scales (VAS vs NRS), follow-up duration, and comparator choice. For example, trials with early endpoints often showed large between-group differences, while those with longer follow-up demonstrated convergence, emphasizing the need for standardized timepoints in future TMD research.

The implications of these findings are clinically significant. A stepwise model emerges: begin with conservative care emphasizing manual therapy plus exercises, then escalate to arthrocentesis when symptoms persist. Device-based modalities may provide adjunctive benefits but appear less robust when evaluated against structured programs. This framework mirrors rehabilitation models in musculoskeletal medicine, where active engagement and procedural escalation are integrated in a tiered fashion.

Despite these encouraging results, several limitations warrant consideration. Methodological diversity among trials - ranging from sample sizes as small as six to seven per arm to larger cohorts exceeding 80 participants - limits direct comparability. Reporting standards were inconsistent, with some studies presenting medians and IQRs converted to means and standard deviations, and others requiring assumptions for sample allocation. Risk of bias also varied; blinding was often absent, and allocation concealment was rarely described in detail. 

Nevertheless, the consistent direction of effect across multiple interventions strengthens the overall conclusion that both conservative and minimally invasive strategies alleviate pain and improve function in TMD. The present synthesis extends prior narrative reviews by quantifying effect sizes across modalities and contextualizing them through subgroup analyses and funnel plot inspection (Figure 5).

Future research should prioritize multicenter RCTs with standardized endpoints and longer follow-up, enabling the evaluation of sustained benefit and relapse rates. Cost-effectiveness analyses are also warranted, given the resource implications of arthrocentesis versus structured physiotherapy. Finally, trials integrating patient-reported outcomes and quality-of-life measures would enhance the relevance of findings to real-world practice.

## Conclusions

The evidence from 10 RCTs indicates that active interventions - particularly manual therapy with exercises and arthrocentesis - consistently outperform passive or standard care in reducing pain and improving function in TMD. While heterogeneity limits definitive ranking of all modalities, the stepwise framework of escalating from multimodal conservative care to arthrocentesis is supported by both pooled results and subgroup analyses.

## References

[1] De Barros Melo MN, Dos Santos Melo JN, Sarmento VA, De Azevedo RA, Queiroz CS (2017). Influence of arthrocentesis irrigation volume at temporomandibular disorder treatment. Indian J Dent Res.

[2] Chellappa D, Thirupathy M (2020). Comparative efficacy of low-level laser and TENS in the symptomatic relief of temporomandibular joint disorders: a randomized clinical trial. Indian J Dent Res.

[3] Urbański P, Trybulec B, Pihut M (2021). The application of manual techniques in masticatory muscles relaxation as adjunctive therapy in the treatment of temporomandibular joint disorders. Int J Environ Res Public Health.

[4] Gębska M, Dalewski B, Pałka Ł, Kołodziej Ł (2023). Evaluation of the efficacy of manual soft tissue therapy and therapeutic exercises in patients with pain and limited mobility TMJ: a randomized control trial (RCT). Head Face Med.

[5] Salloum K, Karkoutly M, Haddad I, Nassar JA (2024). Effectiveness of ultrasound therapy, TheraBite device, masticatory muscle exercises, and stabilization splint for the treatment of masticatory myofascial pain: a randomized controlled trial. Clin Exp Dent Res.

[6] Huang L, Huang Z, Bi S, Mai H (2024). A clinical trial of ropivacaine in arthocentesis for TMD. BMC Oral Health.

[7] Li DT, Luo LY, Li KY, Su YX, Durham J, Leung YY (2024). Early arthrocentesis for temporomandibular joint arthralgia: a superiority trial. Int Dent J.

[8] Mosleh AA (2024). Treatment of temporomandibular joint internal derangement using MESNA injection. BMC Oral Health.

[9] Shah SU, Khan SS, Moin S, Younus S, Jabeen H, Safeer K (2024). Effectiveness of manual therapy, physical therapy in conjunction with patient education for temporomandibular disorders: a randomized controlled study. J Ayub Med Coll Abbottabad.

[10] Tang R, Luo R, Tang S, Song H, Chen X (2022). Machine learning in predicting antimicrobial resistance: a systematic review and meta-analysis. Int J Antimicrob Agents.

[11] Al-Ani Z, Gray R (2007). TMD current concepts: 2. Imaging and treatment options. An update. Dent Update.

[12] Asadpour N, Shooshtari Z, Kazemian M, Gholami M, Vatanparast N, Samieirad S (2022). Combined platelet-rich plasma and hyaluronic acid can reduce pain in patients undergoing arthrocentesis for temporomandibular joint osteoarthritis. J Oral Maxillofac Surg.

[13] Bertolucci LE, Grey T (1995). Clinical analysis of mid-laser versus placebo treatment of arthralgic TMJ degenerative joints. Cranio.

[14] Golanska P, Saczuk K, Domarecka M, Kuć J, Lukomska-Szymanska M (2021). Temporomandibular myofascial pain syndrome - aetiology and biopsychosocial modulation. A narrative review. Int J Environ Res Public Health.

[15] Jayaram P, Kennedy DJ, Yeh P, Dragoo J (2019). Chondrotoxic effects of local anesthetics on human knee articular cartilage: a systematic review. PM R.

[16] Reynolds B, Puentedura EJ, Kolber MJ, Cleland JA (2020). Effectiveness of cervical spine high-velocity, low-amplitude thrust added to behavioral education, soft tissue mobilization, and exercise for people with temporomandibular disorder with myalgia: a randomized clinical trial. J Orthop Sports Phys Ther.

[17] Pessoa DR, Costa DR, Prianti BM, Costa DR, Delpasso CA, Arisawa EÂ, Nicolau RA (2018). Association of facial massage, dry needling, and laser therapy in temporomandibular disorder: case report. Codas.

[18] Tanhan A, Ozer AY, Polat MG (2023). Efficacy of different combinations of physiotherapy techniques compared to exercise and patient education in temporomandibular disorders: a randomized controlled study. Cranio.

[19] Marklund S, Wänman A (2007). Incidence and prevalence of temporomandibular joint pain and dysfunction. A one-year prospective study of university students. Acta Odontol Scand.

[20] Kapos FP, Exposto FG, Oyarzo JF, Durham J (2020). Temporomandibular disorders: a review of current concepts in aetiology, diagnosis and management. Oral Surg.

[21] Dalewski B, Kamińska A, Kiczmer P, Węgrzyn K, Pałka Ł, Janda K, Sobolewska E (2021). Pressure algometry evaluation of two occlusal splint designs in bruxism management - randomized, controlled clinical trial. J Clin Med.

[22] Wan X, Wang W, Liu J, Tong T (2014). Estimating the sample mean and standard deviation from the sample size, median, range and/or interquartile range. BMC Med Res Methodol.

[23] Wu SS, Sun F, Zhan SY (2017). Bias risk assessment: (3) Revised Cochrane bias risk assessment tool for individual randomized, cross-over trials (RoB2.0) (in Chinese). Zhonghua Liu Xing Bing Xue Za Zhi.

[24] Page MJ, McKenzie JE, Bossuyt PM (2021). The PRISMA 2020 statement: an updated guideline for reporting systematic reviews. BMJ.

